# A Engagement Strategy to Build a Dementia Care Infrastructure

**DOI:** 10.1093/geroni/igaf122.604

**Published:** 2025-12-31

**Authors:** Sara Murphy, Jennifer Severance, Matthew Smith

## Abstract

With one of the fastest growing populations in the United States, Texas is home to an increasing number of older adults living with dementia. Guided by the Center for Disease Control and Prevention’s Healthy Brain Initiative (HBI) Road Map, an academic health science center partnered with community, clinical, and public health sectors to engage stakeholders, identify emerging needs, braid together resources and expertise, and develop and strengthen the state’s dementia care infrastructure. This symposium examines multi-sector solutions to improve the quality of care and quality of life for people at risk or living with Alzheimer’s disease and related dementias (ADRD) and their caregivers, help people remain in their homes and communities, and reduce healthcare expenditures. First, presenters introduce a Dementia Consortium of community, health, and public health representatives and people with lived experience that supports dementia-friendly training and education across primary care, long-term care, and community settings. This is followed by results from implementation of an innovative primary care model of dementia navigator services designed to support comprehensive and coordinated care for persons with dementia and to strengthen community-clinical linkages. Finally, presenters explore examples and outcomes of interventions designed to promote brain health, early detection, and diagnosis as well as caregiver health. By applying the HBI public health framework, communities can enhance local collaboration and coordination, raise public and provider awareness about dementia, and design cross-sector strategies to address the healthcare costs and challenges of care for people impacted by ADRD.

